# Modelling Study on Internal Energy Loss Due to Entropy Generation for Non-Darcy Poiseuille Flow of Silver-Water Nanofluid: An Application of Purification

**DOI:** 10.3390/e20110851

**Published:** 2018-11-06

**Authors:** Nasir Shehzad, Ahmed Zeeshan, Rahmat Ellahi, Saman Rashidi

**Affiliations:** 1 Department of Mathematics & Statistics, FBAS, IIUI, Islamabad 44000, Pakistan; 2 Center for Modeling & Computer Simulation, Research Institute, King Fahd University of Petroleum & Minerals, Dhahran 31261, Saudi Arabia; 3 Department of Mechanical Engineering, Ferdowsi University of Mashhad, Mashhad 91775-1111, Iran

**Keywords:** energy loss, silver-water nanofluid, magnetic field, porous media, non-Darcy Poiseuille flow

## Abstract

In this paper, an analytical study of internal energy losses for the non-Darcy Poiseuille flow of silver-water nanofluid due to entropy generation in porous media is investigated. Spherical-shaped silver (Ag) nanosize particles with volume fraction 0.3%, 0.6%, and 0.9% are utilized. Four illustrative models are considered: (i) heat transfer irreversibility (HTI), (ii) fluid friction irreversibility (FFI), (iii) Joule dissipation irreversibility (JDI), and (iv) non-Darcy porous media irreversibility (NDI). The governing equations of continuity, momentum, energy, and entropy generation are simplified by taking long wavelength approximations on the channel walls. The results represent highly nonlinear coupled ordinary differential equations that are solved analytically with the help of the homotopy analysis method. It is shown that for minimum and maximum averaged entropy generation, 0.3% by vol and 0.9% by vol of nanoparticles, respectively, are observed. Also, a rise in entropy is evident due to an increase in pressure gradient. The current analysis provides an adequate theoretical estimate for low-cost purification of drinking water by silver nanoparticles in an industrial process.

## 1. Introduction

Convection in saturated porous media is a popular field of investigation among researchers nowadays because of its numerous applications in painting filtration, microelectronic heat transfer, soil sciences, thermal insulation, petroleum industries, nuclear waste disposal, geothermal systems, chemical catalytic beds, fuel cells, solid matrix heat exchangers, grain storage, etc. Darcy’s law [1], a linear relationship of velocity and pressure gradient, is mathematically expressed by the following relationship:

(1) $$-\nabla \bar{p}=\eta_{1}\bar{u}.$$

It is understood that Darcy’s law is inadequate to describe the high rate of flow in porous media because the low Reynolds number based on the mean pore diameter exceeds 1 to 10. As a matter of fact, when the Reynolds number increases to a critical value or when inertial forces dominate, Equation (1) is not valid anymore and it becomes nonlinear, whereas the structure of nonlinear Darcy’s law for porous media illustrates the mechanism of viscous flow under different geometric and physical conditions. To overcome this deficiency, Forchheimer [2] proposed nonlinear correction of Darcy’s law by the following universal decree:

(2) $$-\nabla \bar{p}=\eta_{1}\bar{u}+\eta_{2}{\bar{u}}^{2},$$

where $\nabla \bar{p}$ is a pressure gradient, $\eta_{1}=\frac{\mu_{nf}}{K}$, and $\eta_{2}$ is an empirical constant in second-order shape related to resistance and represents porosity and pore size [3,4]. 

In addition, low thermal conductivities have gained much attention by researchers in search of higher thermal conductivities for conventional coolants. It is now well accepted that nanofluid offers better thermal efficiency [5] in combinations of nanoparticles (e.g., Cu, Ag, TiO_2_, Al_2_O_3_) with a size of 1–100 nm suspended in carrier fluid (e.g., propylene glycol, kerosene, water, or ethylene glycol) [6,7,8,9,10,11]. In particular, silver nanoparticle is a very effective agent, as seen by its applications in agriculture (fruits, vegetables), medicine (devices, burn treatment, infections [12]), and industry (solar energy absorption, cosmetics, clothing, chemical catalysis, water purification). Silver particles in ionic form exhibit antibacterial action; they are able to break down bacteria such as Escherichia coli and Staphylococcus aureus. Silver nanocolloid in a concentration of 0.8–1.2 ppm removes Escherichia coli bacteria from groundwater. Ceramic filter systems consist of a porous ceramic filter attached to the bottom or top of a plastic or ceramic receptacle. Contaminated water is poured into the top container and passes through the filter into the receptacle below. The lower receptacle usually is fitted with a tap. Ceramic water filter devices can eliminate waterborne pathogens. Currently, such devices are manufactured by pressing and firing a mixture of clay and burnable organic materials like rice husks, flour, and sawdust with silver nanoparticles [13]. The filter is made using a filter press, after which it is air-dried and fired in a kiln. This forms the ceramic material and burns off the sawdust, flour, and rice husks, making the filter porous and permeable to water. Ceramic water filters are also reported to be very effective in removing more than 99% of protozoa and 90–99.99% of bacteria from drinking water [14,15]. It is noted that nanoparticle preparations are very effective in relation to Helicobacter pylori. Silver ions also act synergistically with benzylpenicillin, erythromycin, amoxicillin, and clindamycin [16]. Godson et al. [17] studied the effects of different factors such as temperature (between 323 K and 363 K) and concentration (0.3, 0.6, and 0.9% volume concentration) on the thermal conductivity of Ag-deionized water nanofluid by using uniform nanosized silver particles. Their results showed that thermal conductivity increased 27% to 80% with an increase in temperature and particle concentration from 0.3% to 0.9%. Silver water used in investigations contained antibacterial “silver water” from Nanoco. It was found that exposure of the investigated food material on the activity of the sprayed nanosilver particles could almost double their microbiological and sensorial stability.

Moreover, in the thermodynamics approach, minimization of entropy generation is done to optimize thermal engineering devices for higher energy efficiency. Entropy generation regulates the level of available irreversibility during the process. Consequently, in specific ways, entropy generation measures progress toward thermodynamic equilibrium. It is important to indicate that due to the limitation of first-law efficiency in the heat transfer engineering system, the second law of thermodynamics is more reliable than the first law. Rashidi and Freidoonimehr [18] investigated entropy generation in magnetohydrodynamics (MHD) Hiemenz flow through porous media. They detected increasing entropy generation due to the magnetic parameter and Brinkman number, but the opposite behavior was noted for the case of the Bejan number.

Herein, separate non-Darcy porous media irreversibility (NDI) is discussed in a wavy channel for the first time. Our aim is to indicate the key factors that can be used to control the energy loss (entropy) in said phenomenon. Also, this paper is an attempt to present an adequate theoretical estimate for low-cost purification of drinking water by silver nanoparticles with very low energy loss in an industrial process. More specifically, this work concentrates on MHD mixed convection Poiseuille (different pressure gradient) flow of fluid with silver (Ag) nanoparticles passing through the porous wavy channel. The phenomena of highly coupled nonlinear differential equations are tackled by the homotopic method [19,20,21,22,23,24,25,26,27]. In the subsequent sections, first a mathematical formulation is developed, then the analytical solution, convergence analysis, comprehensive discussion of results, and notable findings are respectively presented and examined through graphs, tables, and bar charts. Finally, the average entropy generation for four different portions—heat transfer irreversibility (HTI), fluid friction irreversibility (FFI), Joule dissipation irreversibility (JDI), and non-Darcy porous media irreversibility (NDI)—are discussed in detail.

## 2. Formulation 

**Figure 1:**
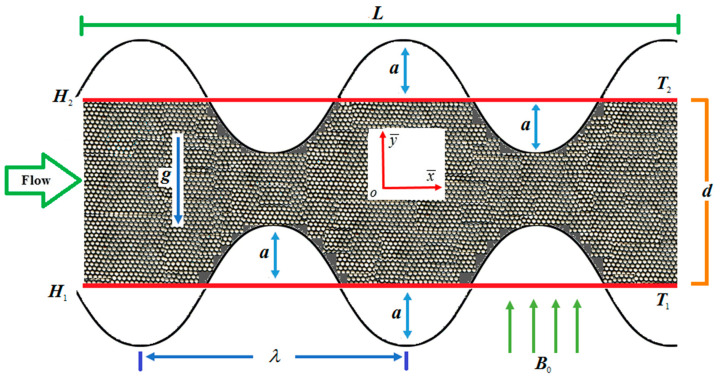
Poiseuille flow model of nanofluid.

Consider two-dimensional (2-D) steady, laminar incompressible viscous nanofluid between two symmetric wavy walls (channels), as displayed in Figure 1. The configuration of the walls with amplitude a, width d, and length L of the channel is defined as:

(3) $$H_{1}=-d-a\cos\left(\frac{2\pi }{L}\bar{x}\right),\;H_{2}=d+a\cos\left(\frac{2\pi }{L}\bar{x}\right).$$

The water-based nanofluid with the suspension of silver nanoparticles is considered. Finally, the proposed model can be expressed as [28,29,30,31,32]:

(4) ∇.V=0,

(5) $$\rho_{nf}\left(V.\nabla \right)V=-\nabla \bar{p}+\mu_{nf}\nabla^{2}V-\frac{\mu_{nf}}{K}V-\rho_{nf}F_{c}\left|V\right|V+{\left(\rho \beta \right)}_{nf}\left(\bar{T}-T_{2}\right)g+J\times B,$$

(6) $${\left(\rho C_{p}\right)}_{nf}\left(V.\nabla \right)\bar{T}=k_{nf}\nabla^{2}\bar{T}+\Phi +\frac{1}{\sigma_{nf}}J.J,$$

where $V,\;\bar{T},\;J,\;B,\;g$ are, respectively, nanofluid velocity, temperature, current density, magnetic field, and gravitational acceleration. 

According to Ohm’s law:

(7) $$J=\sigma_{nf}\left(V\times B\right),$$

where $B=\left[0,B_{0},0\right]$ and $\sigma_{nf}$ is the electrical conductivity of nanofluid.

Under the influence of non-Darcy and magnetic field with mixed convection, Equations (4)–(6) can be obtained as:

(8) $$\frac{\partial \bar{u}}{\partial \bar{x}}+\frac{\partial \bar{u}}{\partial \bar{y}}=0,$$

(9) $$\rho_{nf}\left(\bar{u}\frac{\partial \bar{u}}{\partial \bar{x}}+\bar{v}\frac{\partial \bar{u}}{\partial \bar{y}}\right)=-\frac{\partial \bar{p}}{\partial \bar{x}}+\mu_{nf}\left(\frac{\partial^{2}\bar{u}}{\partial {\bar{x}}^{2}}+\frac{\partial^{2}\bar{u}}{\partial {\bar{y}}^{2}}\right)-\sigma_{nf}B_{0}^{2}\bar{u}-\frac{\mu_{nf}}{K}\bar{u}-\rho_{nf}F_{c}{\bar{u}}^{2}+{\left(\rho \beta \right)}_{nf}g\left(\bar{T}-T_{2}\right),$$

(10) $${\left(\rho C_{p}\right)}_{nf}\left(\bar{u}\frac{\partial \bar{T}}{\partial \bar{x}}+\bar{v}\frac{\partial \bar{T}}{\partial \bar{y}}\right)=k_{nf}\left(\frac{\partial^{2}\bar{T}}{\partial {\bar{x}}^{2}}+\frac{\partial^{2}\bar{T}}{\partial {\bar{y}}^{2}}\right)+{\left(\mu \right)}_{nf}{\left(\frac{\partial \bar{u}}{\partial \bar{y}}\right)}^{2}+\sigma_{nf}B_{0}^{2}{\bar{u}}^{2}.$$

The corresponding boundary conditions can be written in the following form:

(11) $$\begin{array}{l} \bar{u}=0,\;\bar{v}=0,\;\bar{T}=T_{1}\;\mathrm{at}\;\bar{y}=H_{1}\\ \bar{u}=0,\;\bar{v}=0,\;\bar{T}=T_{2}\;\mathrm{at}\;\bar{y}=H_{2}. \end{array}$$

The associated forces for the case of conservation of momentum are as follows:

Inertial term $=\rho_{nf}\left(\bar{u}\frac{\partial \bar{u}}{\partial \bar{x}}+\bar{v}\frac{\partial \bar{u}}{\partial \bar{y}}\right)$, pressure gradient $=-\frac{\partial \bar{p}}{\partial \bar{x}}$, viscous forces $=\mu_{nf}\left(\frac{\partial^{2}\bar{u}}{\partial {\bar{x}}^{2}}+\frac{\partial^{2}\bar{u}}{\partial {\bar{y}}^{2}}\right)$, Lorentz force $=\sigma_{nf}B_{0}^{2}\bar{u}$, non-Darcy forces $=\left(\frac{\mu_{nf}}{K}+\rho_{nf}F_{c}\;\bar{u}\right)\bar{u}$, and convection $={\left(\rho \beta \right)}_{nf}g\left(\bar{T}-T_{2}\right)$, where $B_{0}$ is magnetic field strength and $F_{c}$ is the inertial resistance (coefficient) or Forchheimer correction. 

The terms present in the energy equation can be written as:

Inertial term $={\left(\rho C_{p}\right)}_{nf}\left(\bar{u}\frac{\partial \bar{T}}{\partial \bar{x}}+\bar{v}\frac{\partial \bar{T}}{\partial \bar{y}}\right)$, heat conductivity $=k_{nf}\left(\frac{\partial^{2}\bar{T}}{\partial {\bar{x}}^{2}}+\frac{\partial^{2}\bar{T}}{\partial {\bar{y}}^{2}}\right)$, viscous dissipation $=\mu_{nf}{\left(\frac{\partial \bar{u}}{\partial \bar{y}}\right)}^{2}$, and Joule’s heating $=\sigma_{nf}B_{0}^{2}{\bar{u}}^{2}$.

A new equation for calculating the effective viscosity and thermal conductivity of nanofluids at low volume fractions (0.3, 0.6, and 0.9% volume concentration) and temperature between 323 K and 363 K was proposed by Godson et al. [17] in the following form:

(12) $$\mu_{nf}=\left(1.005+0.497\phi -0.1149\phi^{2}\right)\mu_{f}$$

(13) $$k_{nf}=\left(0.9692\phi +0.9508\right)k_{f}$$

The nanofluid effective density is given by:

(14) $$\rho_{nf}=\left(1-\phi \right)\rho_{f}+\phi \rho_{p}$$

The effective heat capacity of the nanofluid is:

(15) $${\left(\rho C_{p}\right)}_{nf}=\left(1-\phi \right){\left(\rho C_{p}\right)}_{f}+\phi {\left(\rho C_{p}\right)}_{p}$$

The thermal expansion coefficient of the nanofluid is:

(16) $$\beta_{nf}=\frac{\left(1-\phi \right){\left(\rho \beta \right)}_{f}+\phi {\left(\rho \beta \right)}_{p}}{\rho_{nf}}$$

The electrical conductivity of the nanofluid is:

(17) $$\sigma_{nf}=\left[1+\frac{3\left(\frac{\sigma_{p}}{\sigma_{f}}-1\right)\phi }{\left(\frac{\sigma_{p}}{\sigma_{f}}+2\right)-\left(\frac{\sigma_{p}}{\sigma_{f}}-1\right)\phi }\right]\sigma_{f}$$

where ϕ is the solid volume fraction of spherical particles and $C_{p}$ is specific heat. By using the following dimensionless form in Equations (8) and (9):

(18) $$x=\frac{\bar{x}}{\lambda },\;y=\frac{\bar{y}}{d},u=\frac{\bar{u}}{U_{m}},v=\frac{\bar{v}}{U_{m}\delta },\delta =\frac{d}{\lambda },h_{1}=\frac{H_{1}}{d},h_{2}=\frac{H_{2}}{d},p=\frac{d^{2}\bar{p}}{\mu U_{m}\lambda },\theta =\frac{\bar{T}-T_{2}}{T_{1}-T_{2}}.$$

The resulting mathematical model takes the following form:

(19) $$\frac{\partial u}{\partial x}+\frac{\partial v}{\partial y}=0,$$

(20) $$A_{2}Re\delta \left(u\frac{\partial u}{\partial x}+v\frac{\partial u}{\partial y}\right)=A_{1}\left[-\frac{\partial p}{\partial x}+\left(\delta^{2}\frac{\partial^{2}u}{\partial x^{2}}+\frac{\partial^{2}u}{\partial y^{2}}\right)\right]-A_{3}Mu-A_{1}\frac{u}{Da}-A_{2}F^{*}u^{2}+A_{4}Gr\theta ,$$

(21) $$A_{5}RePr\delta \left(u\frac{\partial \theta }{\partial x}+v\frac{\partial \theta }{\partial y}\right)=A_{6}\left(\delta^{2}\frac{\partial^{2}\theta }{\partial x^{2}}+\frac{\partial^{2}\theta }{\partial y^{2}}\right)+A_{3}EcPrMu^{2}+A_{1}EcPr{\left(\frac{\partial u}{\partial y}\right)}^{2},$$

where δ is dimensionless wave number, u and v are velocity components, and θ is dimensionless temperature. 

The velocity component v along the y-axis is considered to be zero due to unidirectional flow along the x-axis, thus Equation (19) eases to $\frac{\partial u}{\partial x}=0$, which indicates that u=u(y). Also, for the case of momentum equation, the *y*-component reduces to $\frac{\partial p}{\partial y}=0$, which means p=p(x) and hence $\frac{\partial p}{\partial x}=P$ (constant). Subsequently, when fluid is flowing due to the constant pressure gradient, then maximum velocity $U_{m}$ will occur between the two walls and will be defined as ($U_{m}=-\frac{a^{2}}{2\mu_{f}}\frac{\partial p}{\partial x}$).

(22) $$\begin{array}{c} Gr=\frac{{\left(\rho \beta \right)}_{f}gd^{2}\left(T_{1}-T_{2}\right)}{\mu_{f}U_{m}},Re=\frac{\rho_{f}U_{m}d}{\mu_{f}},M=\frac{\sigma_{f}{B_{0}}^{2}d^{2}}{\mu_{f}},Da=\frac{K}{d^{2}},\\ F^{*}=\frac{\rho_{f}F_{c}d^{2}U_{m}}{\mu_{f}},Pr=\frac{\mu_{f}{\left(\rho C_{p}\right)}_{f}}{\rho_{f}k_{f}},\;Ec=\frac{U_{m}^{2}}{{\left(C_{p}\right)}_{f}\left(T_{1}-T_{2}\right)},Br=PrEc,\\ A_{1}=\frac{\mu_{nf}}{\mu_{f}},\;A_{2}=\frac{\rho_{nf}}{\rho_{f}},\;A_{3}=\frac{\sigma_{nf}}{\sigma_{f}},\;A_{4}=\frac{{\left(\rho \beta \right)}_{nf}}{{\left(\rho \beta \right)}_{f}},\;A_{5}=\frac{{\left(\rho C_{p}\right)}_{nf}}{{\left(\rho C_{p}\right)}_{f}},\;A_{6}=\frac{k_{nf}}{k_{f}}. \end{array}\}$$

By applying the theory of long wavelength approximation, Equations (19) to (21) become:

(23) $$-A_{1}P+A_{1}\frac{\partial^{2}u}{\partial y^{2}}-A_{3}Mu-A_{1}\frac{u}{Da}-A_{2}F^{*}u^{2}+A_{4}Gr\theta =0$$

(24) $$A_{6}\frac{\partial^{2}\theta }{\partial y^{2}}+A_{3}EcPrMu^{2}+A_{1}EcPr{\left(\frac{\partial u}{\partial y}\right)}^{2}=0.$$

Along the same lines, the corresponding boundary conditions can be achieved as:

(25) $$\begin{array}{l} u=0,\;v=0,\;\theta =1\;\mathrm{at}\;y=h_{1}=-1-\frac{a}{d}\mathrm{Cos}\left(\frac{2\pi \lambda }{L}x\right)\\ u=0,\;v=0,\;\theta =0\;\mathrm{at}\;y=h_{2}=1+\frac{a}{d}\mathrm{Cos}\left(\frac{2\pi \lambda }{L}x\right) \end{array}\}.$$

The significance properties of base fluids and nanoparticles are listed in Table 1, and the values of the different involved ratios $\left(A_{1},A_{2},A_{3},A_{4},A_{5}\;\mathrm{and}\;A_{6}\right)$ are shown in Table 2.

The skin friction coefficient is $C_{f}=\frac{2\tau_{w}}{\rho_{f}{U_{m}}^{2}}$, whereas the walls’ sharing stress can be determined by:

(26) $$\tau_{w}=\mu_{nf}{\left(\frac{\partial \bar{u}}{\partial \bar{y}}\right)}_{\bar{y}=H_{1}\;\mathrm{and}\;H_{2}}.$$

Using the dimensionless variables given in Equation (18), dimensionless skin friction is gained as:

(27) Cf=2A1Reu′(y)|y=h1 and h2.

The Nusselt number is $Nu=\frac{dq_{w}}{k_{f}\left(T_{1}-T_{2}\right)},$ where $q_{w}$ is the heat transfer rate and defined as:

(28) $$q_{w}=-k_{nf}{\left(\frac{\partial \bar{T}}{\partial \bar{y}}\right)}_{\bar{y}=H_{1}\;\mathrm{and}\;H_{2}}.$$

Using Equation (18), the Nusselt number in dimensionless is found as:

(29) $$Nu=-{A_{6}\theta^{\prime }\left(y\right)|}_{y=h_{1}\;\mathrm{and}\;h_{2}}.$$

## 3. Entropy Generation Analysis

For non-Darcy porous media, energy loss due to entropy generation for the case of heat in the presence of a magnetic field is described as:

(30) $$E_{G}=\underset{\begin{array}{c} \mathrm{entropy}\;\mathrm{due}\;\mathrm{to}\\ \mathrm{heat}\;\mathrm{transfer} \end{array}}{\underset{︸}{\frac{k_{nf}}{{T_{2}}^{2}}{\left(\frac{\partial \bar{T}}{\partial \bar{y}}\right)}^{2}}}+\underset{\begin{array}{c} \mathrm{entropy}\;\mathrm{due}\;\mathrm{to}\\ \mathrm{fluid}\;\mathrm{friction} \end{array}}{\underset{︸}{\frac{\mu_{nf}}{T_{2}}{\left(\frac{\partial \bar{u}}{\partial \bar{y}}\right)}^{2}}}+\underset{\begin{array}{c} \mathrm{entropy}\;\mathrm{due}\;\mathrm{to}\\ \mathrm{magnetic}\;\mathrm{field} \end{array}}{\underset{︸}{\frac{\sigma_{nf}{B^{2}}_{0}{\bar{u}}^{2}}{T_{2}}}}+\underset{\begin{array}{c} \mathrm{entropy}\;\mathrm{due}\;\mathrm{to}\;\mathrm{non}-\mathrm{Darcy}\\ \mathrm{porous}\;\mathrm{media} \end{array}}{\underset{︸}{\frac{1}{T_{2}}\left(\frac{\mu_{nf}}{K}+\rho_{nf}F_{c}\;\bar{u}\right){\bar{u}}^{2}}}$$

Equation (30) comprises four parts: the first term on the right-hand side is entropy generation due to the contribution of thermal irreversibility that comprises HTI due to axial conduction from the wavy surface; the second term describes how friction resists the flow; the third term denotes the movement of electrically conducting fluid under the consideration of magnetic field inducing an electric current that circulates in the fluid; and the last one is energy loss due to non-Darcy porous media, which occurs due to the flow rate in porous media. The entropy generation number is similar to the entropy generation rate, which shows the ratio between the local entropy generation rate and the characteristic entropy generation rate $EG_{0}$. Mathematically, one can write it as:

(31) $$EG_{0}=\frac{k_{nf}{\left(T_{1}-T_{2}\right)}^{2}}{d^{2}{T_{2}}^{2}}$$

(32) $$NG=\frac{EG}{EG_{0}}$$

where NG is the dimensional entropy generation:

(33) $$NG=\frac{d^{2}T{*}^{2}}{k_{nf}{\left(T_{1}-T_{2}\right)}^{2}}\times \left[\frac{k_{nf}}{{T_{2}}^{2}}{\left(\frac{\partial \bar{T}}{\partial \bar{y}}\right)}^{2}+\frac{\mu_{nf}}{T_{2}}{\left(\frac{\partial \bar{u}}{\partial \bar{y}}\right)}^{2}+\frac{1}{T_{2}}\left(\frac{\mu_{nf}}{K}+\rho_{nf}F_{c}\;\bar{u}\right){\bar{u}}^{2}\right],$$

hence, the dimensionless entropy generation number NG is obtained as:

(34) $$NG={\left(\frac{\partial \theta }{\partial y}\right)}^{2}+\frac{A_{1}}{A_{6}}\frac{Br}{\Omega }{\left(\frac{\partial u}{\partial y}\right)}^{2}+\frac{A_{3}}{A_{6}}\frac{MBr}{\Omega }u^{2}+\frac{A_{1}}{A_{6}}\frac{Br}{\Omega Da}u^{2}+\frac{A_{2}}{A_{6}}\frac{F^{*}Br}{\Omega }u^{3},$$

where

(35) $$\Omega =\frac{T_{1}-T_{2}}{T_{2}},\;Br=\frac{\mu_{f}U_{m}^{2}}{k_{f}\left(T_{1}-T_{2}\right)}.$$

The dominance of the entropy procedure is essential due to the feebleness of the entropy generation number, so the Bejan number Be is employed to comprehend the possible mechanism. Mathematically, it can be defined as follows:

(36) $$Be=\frac{\mathrm{Entropy}\;\mathrm{generation}\;\mathrm{due}\;\mathrm{to}\;\mathrm{heat}\;\mathrm{transfer}}{\mathrm{Total}\;\mathrm{entropy}\;\mathrm{generation}},\;i.e.,\;Be=\frac{HTI}{HTI+FFI+JDI+NDI},$$

(37) $$HTI={\left(\frac{\partial \theta }{\partial y}\right)}^{2},FFI=\frac{A_{1}}{A_{6}}\frac{Br}{\Omega }{\left(\frac{\partial u}{\partial y}\right)}^{2},JDI=\frac{A_{3}}{A_{6}}\frac{MBr}{\Omega }u^{2},NDI=\frac{A_{1}}{A_{6}}\frac{Br}{\Omega Da}u^{2}+\frac{A_{2}}{A_{6}}\frac{F^{*}Br}{\Omega }u^{3}.$$

In view of Equation (37), Equation (36) becomes:

(38) $$Be=\frac{{\left(\frac{\partial \theta }{\partial y}\right)}^{2}}{\frac{A_{1}}{A_{6}}\frac{Br}{\Omega }{\left(\frac{\partial u}{\partial y}\right)}^{2}+\frac{A_{3}}{A_{6}}\frac{MBr}{\Omega }u^{2}+\frac{A_{1}}{A_{6}}\frac{Br}{\Omega Da}u^{2}+\frac{A_{2}}{A_{6}}\frac{F^{*}Br}{\Omega }u^{3}}.$$

It is understood from Equation (38) that Be∈[0,1]. When the Bejan number = zero, the heat transfer irreversibility is negligible. When the Bejan number < 0.5, irreversibility due to viscous effects dominates. In the case where the Bejan number = 0.5, the sum of fluid friction, Joule dissipation, and non-Darcy porous media irreversibility is double the heat transfer irreversibility. When the Bejan number > 0.5, the entropy due to heat transfer leads to dominance over entropy due to fluid friction, magnetic field, and non-Darcy porous media irreversibility. When the Bejan number = 1, heat transfer irreversibility is equal to the sum of viscous effects. The average entropy generation number can be computed by the following dimensionless relation:

(39) $$NG\_avg=\frac{1}{\forall }\int_{\forall }NG\;d\forall =\frac{1}{\forall }\int_{z}\int_{y}\int_{x}NG\;dx\;dy\;dz,$$

here

(40) $$NG\_avg=\frac{1}{\forall }\int_{h_{1}}^{h_{2}}NG\;dy$$

or

(41) $$NG\_avg=\frac{1}{\forall }\int_{h_{1}}^{h_{2}}\left(HTI+FFI+JDI+NDI\right)\;dy$$

where ∀ denotes the area of geometry. The volume triple integral (Equation (39)) reduces to a line integral due to unidirectional flow. The average energy loss due to entropy generation from fluid flow and heat transfer components can be calculated for a large finite domain, but in this scenario, we obtained average entropy generation in the domain h_1_ and h_2_, as shown by Equation (41).

## 4. Analytic Solution

To get an analytic solution, a homotopic technique [33] is utilized to solve Equations (23) and (24). Initial approximations $u_{0}\left(y\right)$, $\theta_{0}\left(y\right)$ and supplementary linear operators ${£}_{u}$, ${£}_{\theta }$ for velocity and temperature are:

(42) $$\begin{array}{l} u_{0}\left(y\right)=y^{2}-(h_{1}+h_{2})y+(h_{1}h_{2})\\ \theta_{0}\left(y\right)=\frac{y-h_{2}}{h_{1}-h_{2}} \end{array}\}$$

(43) $${£}_{u}=\frac{d^{2}u}{dy^{2}},\;{£}_{\theta }=\frac{d^{2}\theta }{dy^{2}}.\}$$

With convergence control auxiliary parameters $\hbar_{u}$, $\hbar_{\theta }$ and nonlinear operators $N_{u}$, $N_{\theta }$ with embedding parameter ξ∈[0, 1], the homotopy of the zeroth-order problem is written as:

(44) $$\begin{array}{c} \left(1-\xi \right){£}_{u}\left[u\left(y,\xi \right)-u_{0}\left(y\right)\right]=\xi \hbar_{u}N_{u}\left[u\left(y,\xi \right),\;\theta \left(y,\xi \right)\right],\\ \left(1-\xi \right){£}_{\theta }\left[\theta \left(y,\xi \right)-\theta_{0}\left(y\right)\right]=\xi \hbar_{\theta }N_{\theta }\left[u\left(y,\xi \right),\;\theta \left(y,\xi \right)\right]. \end{array}\}$$

(45) $$\begin{array}{c} N_{u}\left[u\left(y,\xi \right),\;\theta \left(y,\xi \right)\right]=-A_{1}P+A_{1}\frac{\partial^{2}u\left(y,\xi \right)}{\partial y^{2}}-A_{3}Mu\left(y,\xi \right)-A_{1}\frac{u\left(y,\xi \right)}{Da}-\\ A_{2}F^{*}u^{2}\left(y,\xi \right)+A_{4}Gr\theta \left(y,\xi \right)\\ N_{\theta }\left[u\left(y,\xi \right),\;\theta \left(y,\xi \right)\right]=A_{6}\frac{\partial^{2}\theta \left(y,\xi \right)}{\partial y^{2}}+A_{3}EcPrHa^{2}u^{2}\left(y,\xi \right)+A_{1}EcPr{\left(\frac{\partial u\left(y,\xi \right)}{\partial y}\right)}^{2} \end{array}\}$$

(46) $$\begin{array}{lll} \mathrm{For} & \xi =0 & \xi =1\\ u\left(y,\xi \right): & u_{0}\left(y\right) & u\left(y\right)\\ \theta \left(y,\xi \right): & \theta_{0}\left(y\right) & \theta \left(y\right) \end{array}\}$$

The solution for velocity and temperature up to the l-th-order approximation can be expressed as:

(47) $$\begin{array}{l} u(y)=u_{0}\left(y\right)+\sum_{k=1}^{l}u_{k}\left(y\right)\\ \theta (y)=\theta_{0}\left(y\right)+\sum_{k=1}^{l}\theta_{k}\left(y\right) \end{array}\}$$

Up to the third-order iteration, analytic expressions of velocity and temperature distributions are obtained as:

(48) $$u\left(y\right)=C_{1}+C_{2}y+C_{3}y^{2}+C_{4}y^{3}+C_{5}y^{4}+C_{6}y^{5}+C_{7}y^{6}+C_{8}y^{7}+C_{9}y^{8}+C_{10}y^{10}.$$

(49) $$\theta \left(y\right)=D_{1}+D_{2}y+D_{3}y^{2}+D_{4}y^{3}+D_{5}y^{4}+D_{6}y^{5}+D_{7}y^{6}+D_{8}y^{7}+D_{9}y^{8}+D_{10}y^{10}.$$

Coefficients $C_{1},C_{2},C_{3},C_{4},C_{5},C_{6},C_{7},C_{8},C_{9},C_{10},D_{1},D_{2},D_{3},D_{4},D_{5},D_{6},D_{7},D_{8},D_{9},D_{10}$ are given in equations of Appendix A.

## 5. Convergence Analysis

The admissible convergence range of both auxiliary parameters $\hbar_{u}$ and $\hbar_{\theta }$ that arises in Equation (47) is very important for an analytic solution. The residual error of velocity $E_{u}$ and temperature distribution $E_{\theta }$ at two successive approximations over embedding parameter ξ∈[0, 1] up to the 25th-order approximations is computed by the following mathematical relations:

(50) $$E_{u}=\sqrt{\frac{1}{26}\sum_{i=0}^{25}{\left(u\left(i/25\right)\right)}^{2}}\;\mathrm{and}\;E_{\theta }=\sqrt{\frac{1}{26}\sum_{j=0}^{25}{\left(\theta \left(j/25\right)\right)}^{2}}.$$

**Figure 2:**
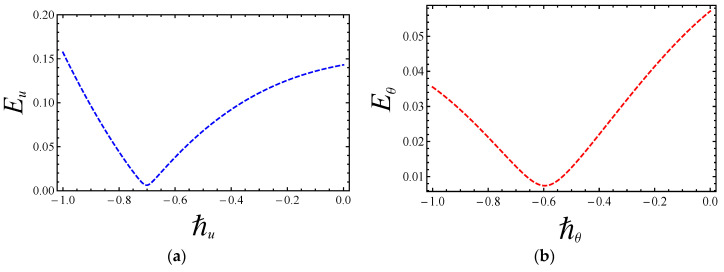
Residual error of (**a**) velocity and (**b**) temperature profiles.

**Figure 3:**
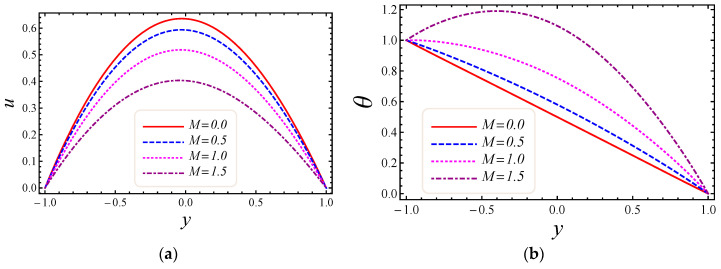
The impact of M on (**a**) velocity and (**b**) temperature profiles.

The above residual formulas give the minimum error for velocity at $\hbar_{u}=-0.7$ and for temperature distribution at $\hbar_{\theta }=-0.6$, which are displayed in Figure 2 and Figure 3, respectively. Table 3 shows residual error for the convergence series solution up to the 25th-order approximation.

## 6. Results and Discussion

**Figure 4:**
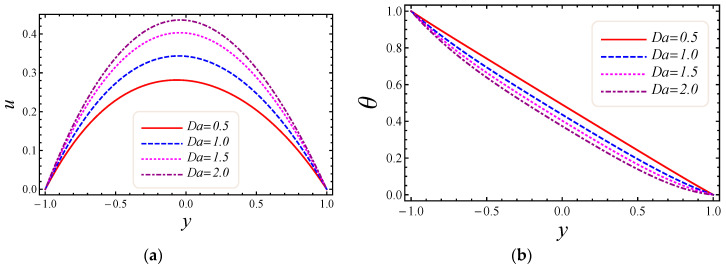
The impact of Darcy number on (**a**) velocity and (**b**) temperature profiles.

**Figure 5:**
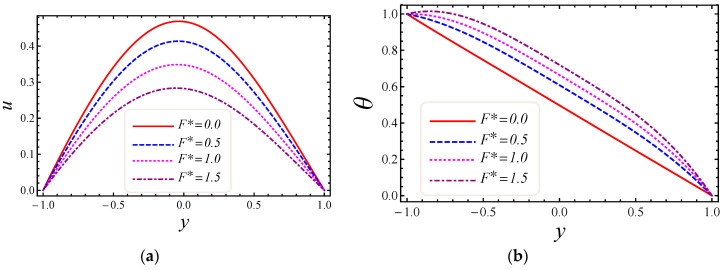
The impact of Forchheimer number on (**a**) velocity and (**b**) temperature profiles.

**Figure 6:**
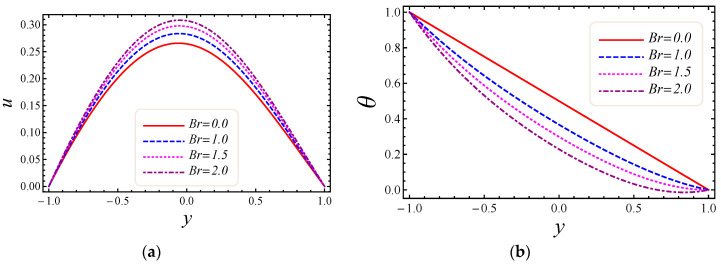
The impact of Brinkman number on (**a**) velocity and (**b**) temperature profiles.

This section describes the role of various parameters on nanoparticle volume fraction, MHD parameter, entropy generation, Darcy number, non-Darcy parameter, Brinkman number, group parameter, Eckert number, Grashof number, Reynolds number, Prandtl number, Bejan number, skin friction, and Nusselt number. Figure 3, Figure 4, Figure 5 and Figure 6 represent the impact of M, Da, F*, and Br on velocity and temperature profiles. Moderately high temperature is used to perform the simulations. The temperature at the upper and lower walls is assumed to be 323 K and 363 K, respectively, in this study. Moreover, high temperature in the range of 323 K to 363 K is used at the inlet section of the channel according to the Godson nanofluid model. In Figure 3a,b, the impact of magnetic field parameter M on velocity and temperature is shown. The Lorentz force is developed by inflicting a vertical magnetic field on the electrically conducting nanofluid. The resultant Lorentz force has the ability to reduce the fluid velocity in confined geometry and causes an increase in temperature. Hence, increasing values of the magnetic field parameter directly affect the increase of thermal boundary-layer thickness, but velocity in the flow direction decreases. In Figure 4a,b, the impact of Darcy number Da on velocity and temperature is elaborated. In Figure 4a, as expected with the increase of Darcy number, the velocity increases, because a higher Darcy number leads to higher permeability of the medium, and with higher permeability the nanofluid can move more easily in the channel. The effect of the Darcy number Da on the dimensionless temperature distribution is depicted in Figure 4b. As is seen, increasing values of the Darcy number lead to smaller values of the dimensionless temperature, which implies that the wall temperature increases rather than the average temperature. The physical explanation is that when the Darcy number increases, fluid velocity in the core of the channel increases significantly (see Figure 4a) so that the energy transferred by fluid convection in this region enhances and then the average temperature decreases. However, the energy transferred by the flow near the wall region is lower because of a slow change in the velocity of this region. Thus, the wall temperature does not vary significantly and it leads to smaller dimensionless temperatures. The performance of the non-Darcy (Forchheimer) number F* on velocity and temperature is shown in Figure 5a,b. It is observed that larger values of the Forchheimer number lead to a stronger thermal boundary layer and weaker momentum boundary layer thickness. In Figure 6a,b, the impact of the Brinkman number Br on velocity and temperature is shown. It can be seen in Figure 6a that the dimensionless velocities increase with increasing Br value. This behavior can be explained by greater thermal energy generated due to the viscous dissipation, which enhances the fluid temperature, and consequently there is a greater buoyancy force. Therefore, an increase in the buoyancy force increases the velocity in the upward direction. In Figure 6b, it is noted that with the increase of dimensionless parameter Br, the dimensionless temperature curves fall, which implies that this parameter increases the wall temperature more than the average temperature. This is due to the fact that very rare energy is transported adjacent to the walls by the fluid flow rather than the core area, which is fallouts of higher values of temperature near the wall area.

**Figure 7:**
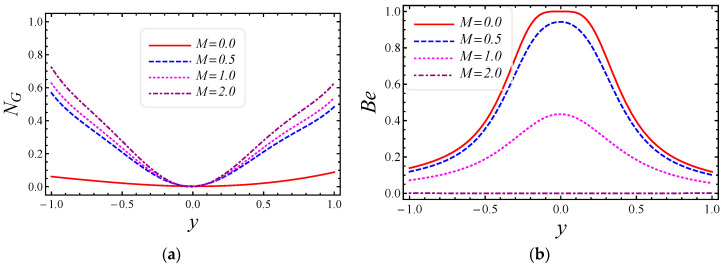
The impact of M on (**a**) entropy generation and (**b**) Bejan number.

**Figure 8:**
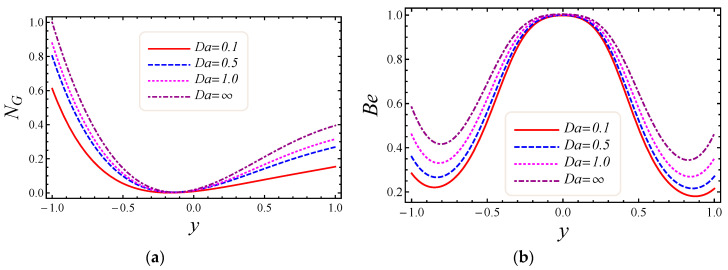
The impact of Darcy number on (**a**) entropy generation and (**b**) Bejan number.

**Figure 9:**
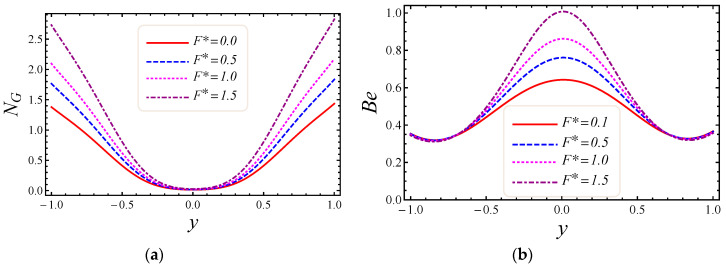
The impact of Forchheimer number on (**a**) entropy generation and (**b**) Bejan number.

**Figure 10:**
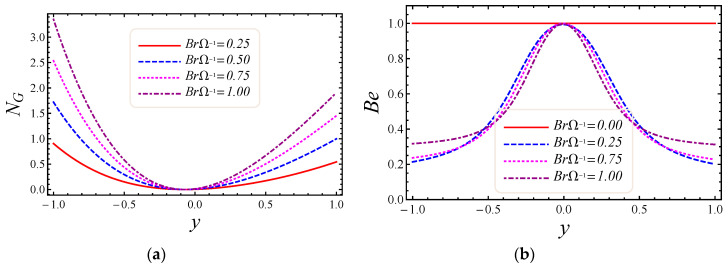
The impact of Br/Ω on (**a**) entropy generation and (**b**) Bejan number.

Figure 7, Figure 8, Figure 9 and Figure 10 represent the impact of M, Da, F*, and Br/Ω on energy loss due to entropy generation and the Bejan number. In Figure 7a,b, the impact of the magnetic field parameter M on entropy generation NG and the Bejan number Be is shown. Energy loss occurs in the system when Lorentz or drag force is created between the fluid and the magnetic field. In Figure 7a, it is perceived that the influence of M on energy loss is maximum at both walls and gradually decreases toward the center of the channel. Energy loss in the middle of the channel is almost zero, so it is detected that M is a major source of energy loss in the system, while the Bejan number gives the dominant decision about fluid friction, magnetic field, and non-Darcy porous media entropy over heat transfer entropy in the system and vice versa. Performance of the magnetic parameter M for silver-water nanofluid on the Bejan number Be is portrayed in Figure 7b. It is noticed that the Bejan number at the center of the channel becomes the maximum value when the magnetic field is neglected. In Figure 8a,b, the impact of the Darcy number Da on entropy generation NG and the Bejan number Be is shown. The permeability of the porous media increases with the increase of Darcy number, thus a large increase in entropy generation is detected at the lower wall as compared to the upper wall, with a large value of Darcy number in Figure 8a. Also, the impact of Da on the Bejan number is displayed in Figure 8b. It is perceived that the Bejan number at the center of the channel attained the extreme value when Da increased. The influence of the non-Darcy (Forchheimer) number F* on entropy generation *N_G_* in Figure 9a and the Bejan number Be in Figure 9b is presented. The same large increment in entropy generation is noticed at both lower and upper walls for different values of F*, but also noticed is that the energy loss is zero at the middle of the channel for all values of the Forchheimer parameter. The Bejan number for various values of the non-Darcy (Forchheimer) parameter F* can be observed in Figure 9b. It is found that for the Forchheimer number, the Bejan number near the middle of the channel increases with the corresponding values of F*. In Figure 10a,b, controlling the effects of the Brinkman number Br/Ω on energy loss due to entropy generation and the Bejan number Be is observed. As entropy generation is a function of the group parameter Br/Ω, it contains the ratio of Brinkman number Br and dimensionless temperature difference $\Omega =\left(T_{1}-T_{2}\right)/T_{2}$. The behavior of Br/Ω when Br=2 and a mixed convection parameter Gr=0.5 on entropy generation is shown in Figure 10a, which describes that increasing values of group parameter cause an enhancement of the buoyancy force in the system, and in response to this a large increase in entropy generation is detected at the lower wall as compared to the upper wall. The result of the group parameter with Br=2 and Gr=0.5 on the Bejan number is clearly elaborated in Figure 10b. The Bejan number attains its maximum value 1 at y=0.2 due to an increase in heat transfer irreversibility with the absence of the group parameter, but gradually decreases and has a value less than 1 toward both walls. This energy loss only occurs due to fluid heat transfer in a particular cross-section of the channel. Non-Darcy porous media irreversibility is introduced in average entropy generation for the first time. 

**Figure 11:**
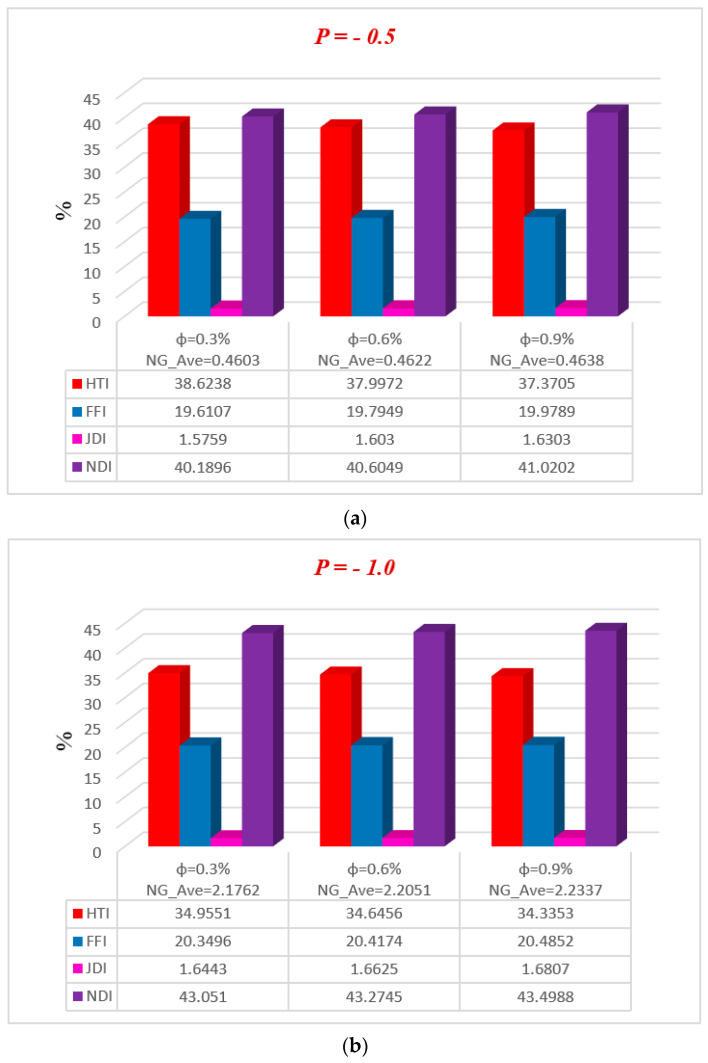
Average breakdown in entropy generation for different ϕ at (**a**) P=−0.5 and (**b**) P=−1.0.

**Figure 12:**
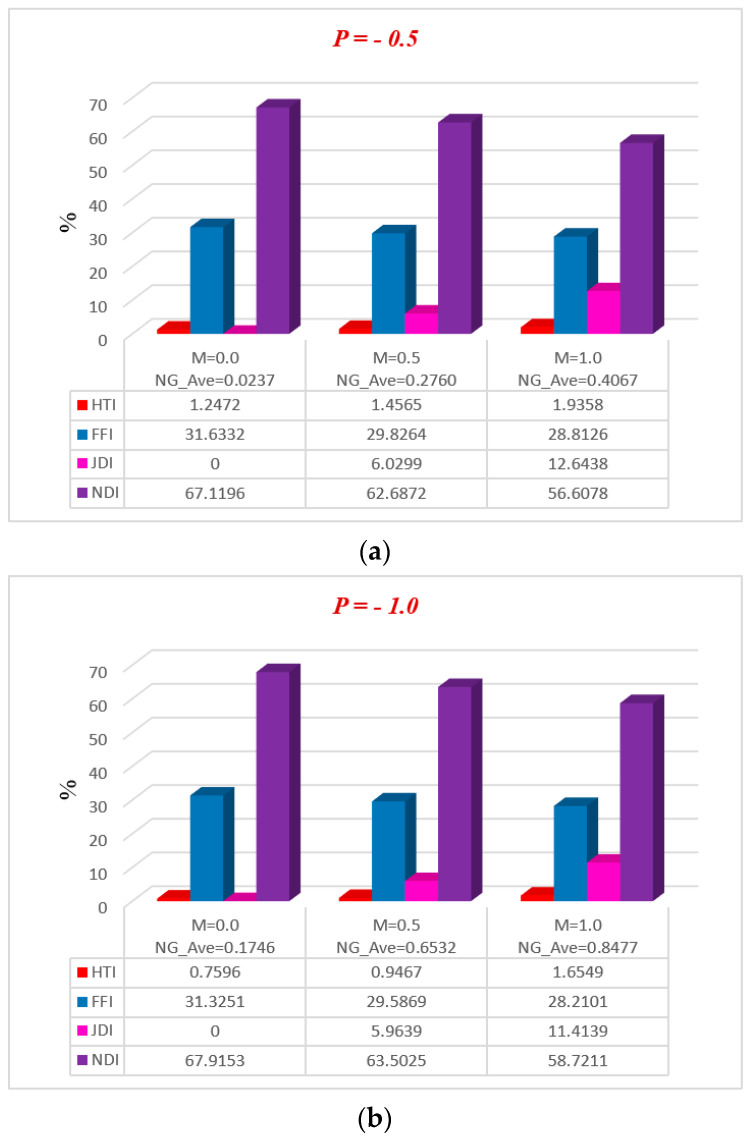
Average breakdown in entropy generation due to M at (**a**) P=−0.5 and (**b**) P=−1.0.

**Figure 13:**
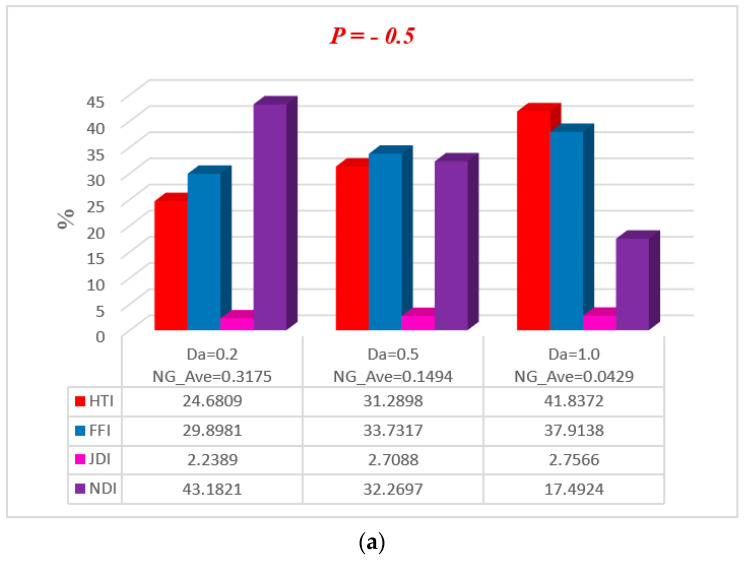

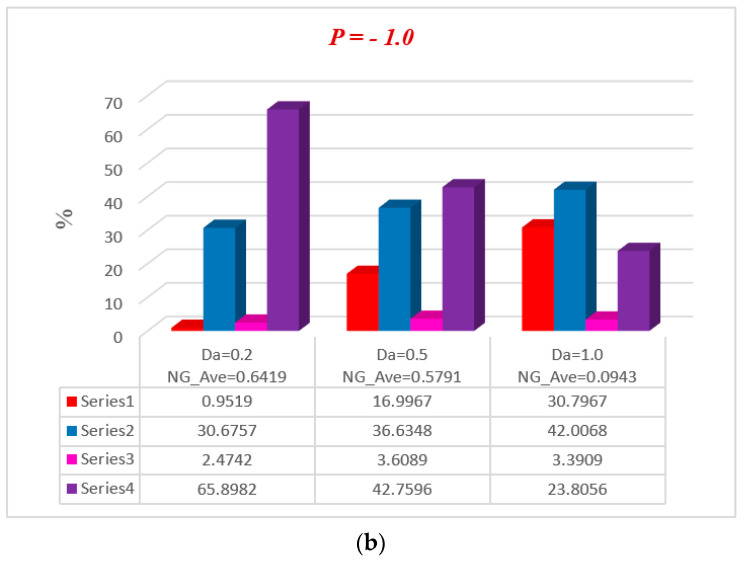
Average breakdown in entropy generation due to Da at (**a**) P=−0.5 and (**b**) P=−1.0.

**Figure 14:**
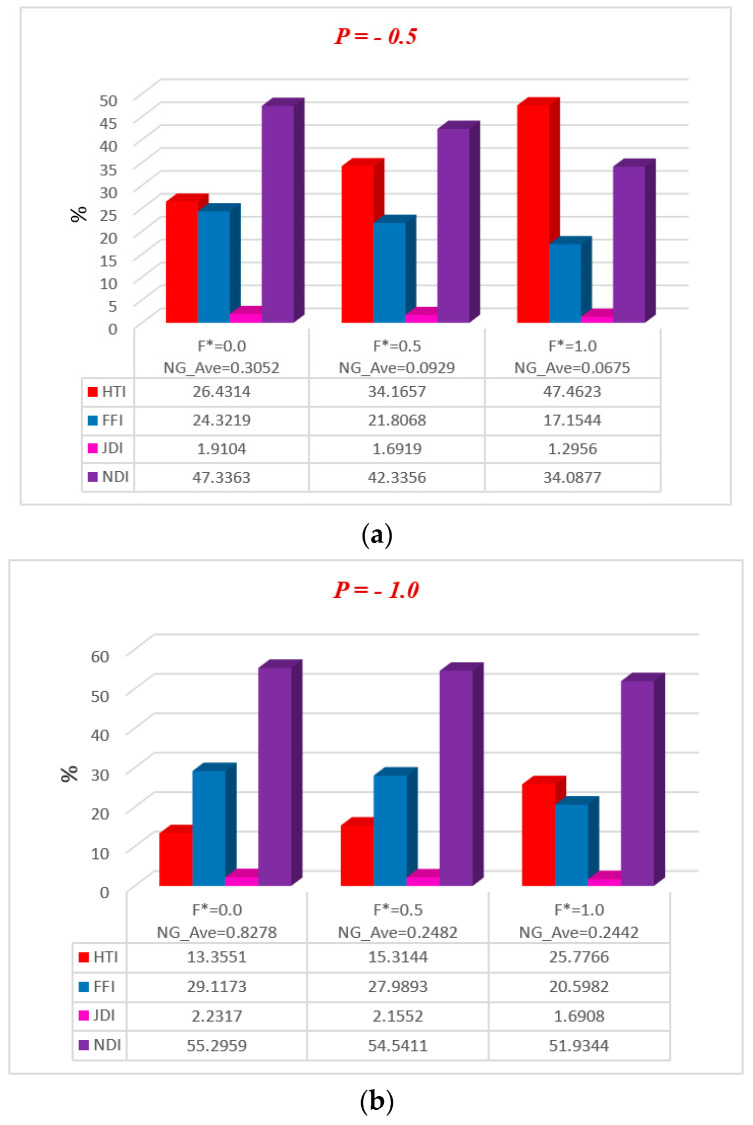
Average breakdown in entropy generation due to F* at (**a**) P=−0.5 and (**b**) P=−1.0.

**Figure 15:**
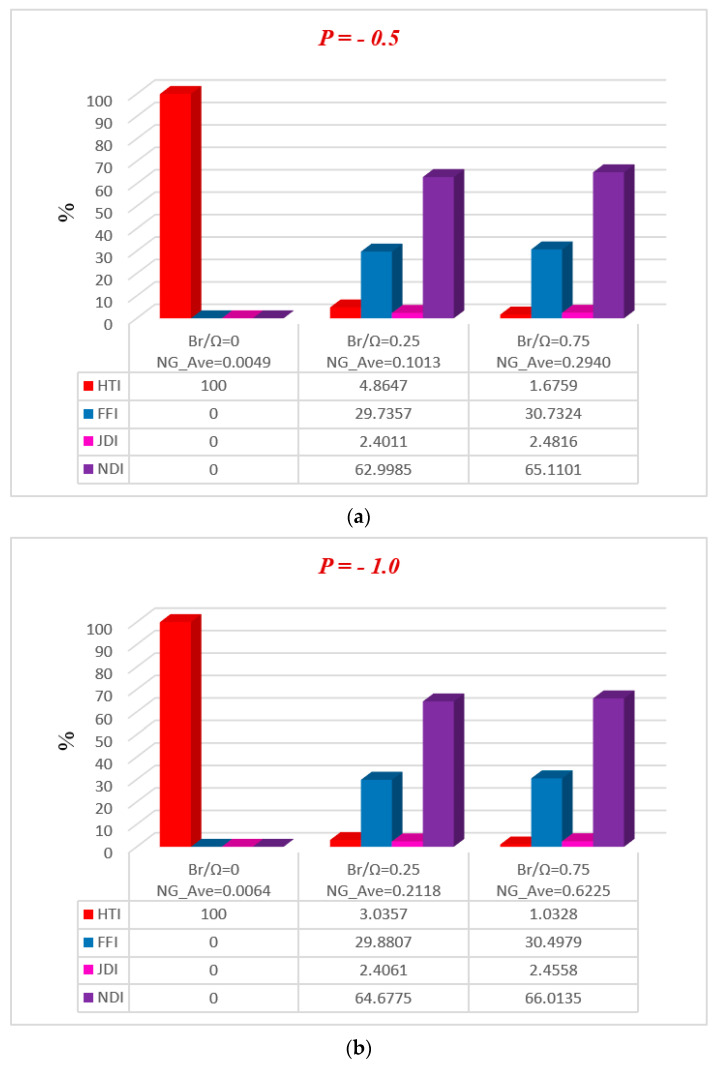
Average breakdown in entropy generation for Br/Ω at (**a**) P=−0.5 and (**b**) P=−1.0.

Figure 11, Figure 12, Figure 13, Figure 14 and Figure 15 represent, in bar charts, the impact of ϕ, M, Da, F*, and Br/Ω on average energy loss due to entropy generation. These bar charts are drawn at different pressure gradients (P=−0.5 and P=−1.0). In Figure 11a,b, it can be seen that the average entropy at both pressure gradients is gradually reduced with the increase of nanoparticle volume fraction ϕ. In the case of a low concentration of silver nanoparticle sustained in the base fluid, when ϕ=0.3%, the average entropy of the whole system is 0.4603 at P=−0.5 and 2.1762 at P=−1.0. Gradually, when the concentration of silver nanoparticles increases in the base fluid, it is clearly observed that the average energy loss due to entropy generation is increased. Nanoparticle concentration directly affects the fluid friction, Joule dissipation, and non-Darcy irreversibility, therefore FFI, JDI, and NDI are increased with the increase of ϕ. The average breakdown in entropy generation due to MHD directly affects Joule dissipation irreversibility, as shown in Figure 12a,b. It is seen in both figures that when the magnetic parameter M is zero, the Joule dissipation irreversibility vanishes, but as the magnetic parameter increases its values, the Joule dissipation irreversibility boosts up speedily. It is also noted that fluid friction irreversibility is reduced for large values of the magnetic parameter at different pressure gradients. Non-Darcy porous media irreversibility depends on the Darcy number Da and the non-Darcy (Forchheimer) parameter F*, as shown in Figure 13a,b and Figure 14a,b. The Darcy number gives the opposite behavior of its increasing values via NDI. As the Darcy number increases, the average entropy and non-Darcy porous media irreversibility of the system decrease, while fluid friction, heat transfer, and Joule dissipation irreversibility boost up quickly for both pressure gradient cases. However, in Figure 14a,b, the non-Darcy (Forchheimer) parameter F* gives the same trend for non-Darcy porous media irreversibility as the Darcy number in Figure 13a,b, because when the Darcy number is large, the flow tends to behave as a non-Darcy flow. For Br=1, the variation of four group parameters Br/Ω on average entropy generation is shown in Figure 15a,b. It is observed that the when the group parameter Br/Ω=0, 100% entropy loss occurs in heat transfer irreversibility, while there is no entropy loss in fluid friction, Joule dissipation, and non-Darcy porous media irreversibility. Moreover, as the group parameter increases in the system, the heat transfer irreversibility decreases while the fluid friction, Joule dissipation, and non-Darcy porous media irreversibility increase progressively; it is also noted that average entropy is directly proportional to group parameter. The magnitude of the average entropy generation rate is higher for higher values of Br/Ω. The effects of emerging parameters are presented in Table 4 and Table 5. It can be seen from calculations that skin friction at the lower and upper walls decreases with the increase of Darcy number (Da) and non-Darcy (Forchheimer) parameter (F*), while the Nusselt number increases at the lower wall, but the reduction is shown at the upper wall. Similar results for the Grashof number (Gr) and Brinkman number (*Br*) are deducted on the Nusselt number at both walls, but skin friction decreases at the lower wall while increasing at the upper. The behavior of $C_{f}$ (skin friction) and Nu (Nusselt number) via magnetic field parameter *M* and the particle volume fraction ϕ are revealed in Table 6 and Table 7, respectively. The prominent increase in volume fraction of nanoparticles and magnetic field parameter is noticed, whereas Nusselt number and skin friction coefficients decrease at the lower wall, while the opposite trend occurs at the upper wall. The thermal conductivity and effective viscosity of silver-water nanofluids increase with the increase in particle volume concentrations of 0.3%, 0.6%, and 0.9%. The existing old correlations for thermal conductivity and viscosity of nanofluids give lower values as compared to new correlations for the properties proposed by Godson et al. [17]. It is also observed that the thermal conductivity enhancement is higher than the viscosity enhancement for the same volume concentration.

## 7. Conclusions

In this paper, energy loss due to entropy generation for the non-Darcy porous media Poiseuille (different pressure gradient) flow of nanofluid through a wavy channel is analyzed. The continuity, momentum, energy, and entropy generation equations are transformed by using a similarity transformation to obtain nonlinear Ordinary differential equations (ODEs). Homotopy analysis method (HAM) is used to solve the nonlinear ODEs subject to the boundary conditions. Results of nanoparticle volume fraction, magnetic field parameter, Darcy number, non-Darcy (Forchheimer) parameter, Brinkman number, entropy generation, Bejan number, skin friction, Nusselt number, and average energy loss due to entropy generation on velocity and temperature were determined numerically as well as graphically by using Mathematica software. The major findings investigated during the study are as follows:

- It is noticed that velocity gives the reduction flow map with increasing values of magnetic field and non-Darcy (Forchheimer) parameter, while velocity increases for large values of Darcy and Brinkman number.
- Temperature distribution increases for increasing values of M and non-Darcy (Forchheimer) F*. On the other hand, the temperature profile decreases for various values of Darcy Da and Brinkman number Br.
- Energy loss due to entropy generation becomes stronger along the walls of the channel for the magnetic field M and non-Darcy (Forchheimer) parameter F*, and near the center of the channel energy loss becomes zero for said parameters.
- Energy loss due to entropy generation becomes weaker at the upper wall as compared to the lower wall of the channel for Darcy number Da, and group parameter Br/Ω is also negligible near the middle of the channel.
- The Bejan number at the center of the channel attained maximum value when the magnetic field was neglected, and Be gained extreme value when group parameter was zero. Moreover, the Bejan number accelerated at boundaries with a large value of Darcy number and at the center of the channel increased with non-Darcy (Forchheimer) parameter.
- Non-Darcy porous media irreversibility in the average break of energy loss due to entropy generation was enhanced with enhancing nanoparticle volume fraction ϕ, non-Darcy (Forchheimer) parameter F*, and group parameter Br/Ω, but the reduction in non-Darcy porous media irreversibility was due to magnetic field parameter M and Darcy number Da.
- A rise in entropy was evident due to an increase in the pressure gradient.

## Figures and Tables

**Table 1:** Physical properties of water and nanoparticles [13].

Property	Water (H_2_O)	Silver (Ag)
$\rho \left(Kg/m^{3}\right)$	9.877 × 10^2^	10,500
$C_{p}\left(J/kg.K\right)$	4.066 × 10^3^	235
$\sigma \left(m^{-1}\right)$	5.0 × 10^−2^	6.30 × 10^7^
k(W/mK)	6.44 × 10^−1^	429

**Table 2:** Parametric values of physical nanofluid for different volume fractions.

ϕ	$A_{1}$	$A_{2}$	$A_{3}$	$A_{4}$	$A_{5}$	$A_{6}$
0.3%	1.0065	1.0286	0.0090	0.9998	0.9988	0.9537
0.6%	1.0080	1.0572	0.0181	0.9997	0.9976	0.9566
0.9%	1.0095	1.0858	0.0272	0.9995	0.9963	0.9595

**Table 3:** Residual error of series solutions when Gr=0.5, Br=1, F*=1, Da=2, and M=0.2.

Order of Approximation	Time	$E_{u}$	$E_{\theta }$
05	8.2651	1.3340 × 10^−3^	2.3980 × 10^−3^
10	35.1732	7.4001 × 10^−5^	3.2385 × 10^−6^
15	67.9793	1.5624 × 10^−8^	3.5705 × 10^−9^
20	187.6291	1.6199 × 10^−12^	4.7723 × 10^−14^
25	296.1218	1.7193 × 10^−16^	1.7037 × 10^−17^

**Table 4:** Effect of Darcy number (Da) and non-Darcy (Forchheimer) parameter on Nu and $C_{f}$ when Gr=0.5, Br=1, M=0.5, and ϕ=0.3%.

Da	F*	Nu(−1)	Nu(1)	$C_{f}\left(-1\right)$	$C_{f}\left(1\right)$
0.5	0.0	0.3111	0.6329	1.3898	−1.069
	0.5	0.3836	0.5634	1.2572	−0.9680
	1.0	0.3928	0. 5483	1.0021	−0.7155
	1.5	0.5067	0. 4428	0.7424	−0.4595
1.0	0.0	0.6497	0.3028	1.5772	−1.2715
	0.5	0.6854	0.2655	1.5600	−1.2552
	1.0	0.8509	0.0987	1.6243	−1.3209
	1.5	1.1791	−0.2298	1.9714	−1.6666
2.0	0.0	0.7814	0.1738	1.7266	−1.4126
	0.5	0.9048	0.0496	1.8225	−1.5089
	1.0	1.1602	−0.2057	2.1024	−1.7891
	1.5	1.5454	−0.5894	2.7338	−2.4202
10.0	0.0	0.9064	0.0517	1.8619	−1.5410
	0.5	1.0892	−0.1309	2.0655	−1.7442
	1.0	1.3881	−0.4285	2.5071	−2.1850
	1.5	1.7725	−0.8098	3.2985	−2.9743

**Table 5:** Effect of Grashof number (Gr) and Brinkman number (Br) on Nu and $C_{f}$ when Da=10, F*=1, M=0.5, and ϕ=0.3%.

Gr	Br	Nu(−1)	Nu(1)	$C_{f}\left(-1\right)$	$C_{f}\left(1\right)$
0.2	0	0.4768	0.4768	1.9264	−1.8003
	1	1.1824	−0.2266	1.8695	−1.7435
	2	1.8639	−0.9062	1.8104	−1.6846
	3	2.5192	−1.5601	1.7491	−1.6234
0.5	0	0.4768	0.4768	2.2683	−1.9541
	1	1.1602	−0.2057	2.1024	−1.7891
	2	1.7575	−0.8043	1.9234	−1.6110
	3	2.2464	−1.3067	1.7315	−1.4199
0.7	0	0.4768	0.4768	2.4907	−2.0519
	1	1.1301	−0.1795	2.2375	−1.8004
	2	1.6391	−0.6957	1.9601	−1.5246
	3	1.9802	−1.0481	1.6583	−1.2244
1.0	0	0.4768	0.4768	2.8159	−2.1914
	1	1.0622	−0.1220	2.4125	−1.7912
	2	1.3923	−0.4735	1.9632	−1.3449
	3	1.4183	−0.5289	1.4679	−0.8527

**Table 6:** Variation of $C_{f}$ for nanoparticle volume fraction and magnetic field parameter when Da=2, F*=1, Gr=0.5, and Br=1.

ϕ	M	Einstein [34]		Godson et al. [17]		Absolute Error	
		$C_{f}\left(-1\right)$	$C_{f}\left(1\right)$	$C_{f}\left(-1\right)$	$C_{f}\left(1\right)$	At Lower Wall	At Upper Wall
0.0%	0.5	2.1334	−1.8122	2.1247	−1.8050	0.0087	0.0072
	1.0	0.8328	−0.5086	0.8404	−0.5157	0.0076	0.0071
	1.5	−1.4790	1.8133	−1.4429	1.7756	0.0361	0.0377
	2.0	−4.7799	5.1249	−4.6860	5.0293	0.0939	0.0956
0.3%	0.5	2.1395	−1.8206	2.1419	−1.8227	0.0024	0.0021
	1.0	0.8417	−0.5175	0.8461	−0.5217	0.0044	0.0042
	1.5	−1.4898	1.8224	−1.4752	1.8081	0.0146	0.0143
	2.0	−4.8487	5.1925	−4.8034	5.1474	0.0453	0.0451
0.6%	0.5	2.1453	−1.8287	2.1601	−1.8414	0.0148	0.0127
	1.0	0.8485	−0.5265	0.8523	−0.5281	0.0038	0.0016
	1.5	−1.5010	1.8318	−1.5088	1.8418	0.0078	0.0100
	2.0	−4.9202	5.2628	−4.9270	5.2718	0.0068	0.0090
0.9%	0.5	2.1508	−1.8364	2.1791	−1.8608	0.0283	0.0244
	1.0	0.8554	−0.5354	0.8590	−0.5350	0.0036	0.0004
	1.5	−1.5125	1.8416	−1.5438	1.8770	0.0313	0.0354
	2.0	−4.9945	5.3359	−5.0571	5.4026	0.0626	0.0667

**Table 7:** Variation of Nu for nanoparticle volume fraction and magnetic field parameter when Da=2, F*=1, Gr=0.5, and Br=1.

ϕ	M	Maxwell Model [35]		Godson et al. [17]		Absolute Error	
		Nu(−1)	Nu(1)	Nu(−1)	Nu(1)	At Lower Wall	At Upper Wall
0.0%	0.5	1.2131	−0.1831	1.2367	−0.2059	0.0236	0.0228
	1.0	1.2806	−0.2283	1.2991	−0.2457	0.0185	0.0174
	1.5	0.2233	0.8759	0.2110	0.8901	0.0123	0.0142
	2.0	−5.4104	6.5961	−5.5297	6.7189	0.1193	0.1228
0.3%	0.5	1.2166	−0.1873	1.2528	−0.2221	0.0362	0.0348
	1.0	1.2924	−0.2405	1.3144	−0.2606	0.0220	0.0201
	1.5	0.2174	0.8820	0.1745	0.9279	0.0429	0.0459
	2.0	−5.5766	6.7639	−5.8279	7.0202	0.2513	0.2563
0.6%	0.5	1.2199	−0.1913	1.2693	−0.2388	0.0494	0.0475
	1.0	1.3042	−0.2528	1.3297	−0.2757	0.0255	0.0229
	1.5	0.2109	0.8886	0.1353	0.9683	0.0756	0.0797
	2.0	−5.7487	6.9375	−6.1410	7.3365	0.3923	0.3990
0.9%	0.5	1.2230	−0.1951	1.2863	−0.2558	0.0633	0.0607
	1.0	1.3161	−0.2652	1.3452	−0.2909	0.0291	0.0257
	1.5	0.2040	0.8957	0.09331	1.0115	0.1107	0.1158
	2.0	−5.9269	7.1172	−6.4696	7.6682	0.5427	0.5510

## Appendix A

Coefficients of polynomial Equation (48):

$$\begin{array}{l} C_{1}=-1-3A_{1}\hbar_{u}-\frac{5A_{1}}{6Da}\hbar_{u}+\frac{11A_{2}F*}{15}\hbar_{u}-\frac{A_{4}Gr}{2}\hbar_{u}+\frac{7A_{1}A_{2}BrGr}{45}\hbar_{u}\hbar_{\theta }-\frac{3A_{1}^{2}}{2}\hbar_{u}^{2}-\frac{61A_{1}^{2}}{360Da^{2}}\hbar_{u}^{2}-\\ \frac{25A_{1}^{2}\hbar_{u}^{2}}{24Da}+\frac{22A_{1}A_{2}F*\hbar_{u}^{2}}{15}+\frac{25A_{1}A_{2}F*\hbar_{u}^{2}}{56Da}-\frac{4919A_{2}^{2}F*\hbar_{u}^{2}}{18900}-\frac{A_{1}A_{4}Gr\hbar_{u}^{2}}{4}-\frac{5A_{1}A_{4}Gr\hbar_{u}^{2}}{48Da}+A_{3}M^{2}\hbar_{u}^{2}\\ \frac{11A_{2}A_{4}F*Gr\hbar_{u}^{2}}{60}+\frac{739A_{3}A_{4}BrGrM^{2}}{5040}\hbar_{u}\hbar_{\theta }+\frac{A_{1}A_{3}M^{2}\hbar_{u}^{2}}{2}+\frac{5A_{1}A_{3}M^{2}}{24Da}\hbar_{u}^{2}-\frac{11A_{2}A_{3}F*M^{2}}{30}\hbar_{u}^{2}, \end{array}$$

$$C_{2}=\frac{1}{6}A_{4}Gr\hbar_{u}+\frac{1}{12}A_{1}A_{4}Gr\hbar_{u}^{2}+\frac{7A_{1}A_{4}Gr}{720Da}\hbar_{u}^{2}-\frac{19A_{2}A_{4}F*Gr}{1260}\hbar_{u}^{2},$$

$$\begin{array}{l} C_{3}=1+3A_{1}\hbar_{u}+\frac{A_{1}}{Da}\hbar_{u}-A_{2}F*\hbar_{u}+\frac{1}{2}A_{4}Gr\hbar_{u}-\frac{1}{6}A_{1}A_{4}BrGr\hbar_{u}\hbar_{\theta }+\frac{3}{2}A_{1}^{2}\hbar_{u}^{2}+\frac{5A_{1}^{2}}{24Da^{2}}\hbar_{u}^{2}-\\ \frac{5A_{1}^{2}}{4Da}\hbar_{u}^{2}-2A_{1}A_{2}F*\hbar_{u}^{2}-\frac{3A_{1}A_{2}F*}{5Da}\hbar_{u}^{2}+\frac{11}{30}A_{2}^{2}F{*}^{2}\hbar_{u}^{2}+\frac{1}{4}A_{1}A_{4}Gr\hbar_{u}^{2}+\frac{1}{4}A_{1}A_{4}F*Gr\hbar_{u}^{2}-\\ A_{3}M^{2}F*\hbar_{u}-\frac{11}{60}A_{3}A_{4}BrGrM\hbar_{u}\hbar_{\theta }-\frac{1}{2}A_{1}A_{3}M^{2}\hbar_{u}^{2}-\frac{1}{4Da}A_{1}A_{3}M^{2}\hbar_{u}^{2}+\frac{1}{2}A_{2}A_{3}F*M^{2}\hbar_{u}^{2}, \end{array}$$

$$C_{4}=-\frac{1}{6}A_{4}Gr\hbar_{u},\;$$

$$\begin{array}{l} C_{5}=-\frac{A_{1}^{2}}{24Da}\hbar_{u}^{2}-\frac{5A_{1}^{2}}{24Da}\hbar_{u}^{2}-\frac{2}{3}A_{1}A_{2}F*\hbar_{u}^{2}+\frac{7A_{1}A_{2}F*}{36Da}\hbar_{u}^{2}-\frac{13}{90}A_{2}^{2}F{*}^{2}\hbar_{u}^{2}-\frac{A_{1}A_{4}Gr}{48Da}\hbar_{u}^{2}+\\ \frac{1}{12}A_{4}A_{2}F*Gr\hbar_{u}^{2}+\frac{1}{24}A_{3}A_{4}BrGrM^{2}\hbar_{u}\hbar_{\theta }+\frac{1}{24Da}A_{1}A_{3}M^{2}\hbar_{u}^{2}-\frac{1}{6}A_{3}A_{2}F*M^{2}\hbar_{u}^{2}, \end{array}$$

$$C_{6}=\frac{A_{4}A_{1}Gr}{240Da}\hbar_{u}^{2}-\frac{1}{60}A_{4}A_{2}F*Gr\hbar_{u}^{2};$$

$$\begin{array}{l} C_{7}=A_{2}F*\hbar_{u}+\frac{1}{90}A_{1}A_{4}BrGr\hbar_{u}\hbar_{\theta }+\frac{A_{1}^{2}}{360Da^{2}}\hbar_{u}^{2}-\frac{2}{15}A_{1}A_{2}F*\hbar_{u}^{2}-\frac{2A_{1}A_{2}F*}{45Da}\hbar_{u}^{2}+\frac{2A_{2}^{2}}{45}F{*}^{2}\hbar_{u}^{2}-\\ \frac{1}{60}A_{4}A_{2}F*\hbar_{u}^{2}-\frac{1}{180}A_{3}A_{4}GrBrM^{2}\hbar_{u}\hbar_{\theta }+\frac{1}{30}A_{3}A_{2}F*M^{2}\hbar_{u}^{2}; \end{array}$$

$$\begin{array}{l} C_{8}=\frac{1}{252}A_{4}A_{2}F*Gr\hbar_{u}^{2},\;C_{9}=\frac{A_{1}A_{2}F*}{280Da}\hbar_{u}^{2}-\frac{A_{2}^{2}}{140}F{*}^{2}\hbar_{u}^{2}+\frac{1}{1680}A_{3}A_{4}BrGrM^{2}\hbar_{u}\hbar_{\theta },\\ C_{10}=\frac{A_{2}^{2}}{1350}F{*}^{2}\hbar_{u}^{2}. \end{array}$$

Coefficients of polynomial Equation (49):

$$\begin{array}{l} D_{1}=\frac{1}{2}-\frac{2}{3}A_{1}Br\hbar_{\theta }-\frac{1}{3}A_{1}A_{6}Br\hbar_{\theta }^{2}-\frac{13A_{1}^{2}Br}{45Da}\hbar_{u}\hbar_{\theta }+\frac{163A_{1}A_{2}BrF*}{630}\hbar_{u}\hbar_{\theta }-\frac{11}{55}A_{3}BrM^{2}\hbar_{u}-\\ \frac{1}{6}A_{1}A_{4}BrGr\hbar_{u}\hbar_{\theta }-\frac{11}{30}A_{3}A_{6}BrM^{2}\hbar_{\theta }^{2}-\frac{23}{30}A_{1}A_{3}BrM^{2}\hbar_{u}\hbar_{\theta }-\frac{1511}{5040Da}A_{1}A_{3}BrM^{2}\hbar_{u}\hbar_{\theta }+\\ A_{1}^{2}Br\hbar_{u}\hbar_{\theta }+\frac{4919}{18900}A_{2}A_{3}BrF*M^{2}\hbar_{u}\hbar_{\theta }-\frac{11}{60}A_{4}A_{3}BrGrM^{2}\hbar_{u}\hbar_{\theta }+\frac{11}{30}A_{3}^{2}BrM^{2}\hbar_{u}\hbar_{\theta }, \end{array}$$

$$D_{2}=-\frac{1}{2}+\frac{1}{180}A_{1}A_{4}BrGrM^{2}\hbar_{u}\hbar_{\theta }+\frac{19}{1260}A_{3}A_{4}BrGrM^{2}\hbar_{u}\hbar_{\theta },$$

$$\begin{array}{l} D_{3}=+A_{3}BrM^{2}\hbar_{\theta }+\frac{1}{2}A_{3}A_{6}BrM^{2}\hbar_{\theta }^{2}+\frac{3}{2}A_{3}A_{1}BrM^{2}\hbar_{u}\hbar_{\theta }+\frac{5}{12Da}A_{3}A_{1}BrM^{2}\hbar_{u}\hbar_{\theta }-\\ \frac{11}{30}A_{3}A_{2}BrF*M^{2}\hbar_{u}\hbar_{\theta }+\frac{1}{4}A_{3}A_{4}BrGrM^{2}\hbar_{u}\hbar_{\theta }-\frac{1}{2}A_{3}^{2}BrM^{4}\hbar_{1}\hbar_{2}, \end{array}$$

$$D_{4}=\frac{1}{18}A_{1}A_{4}BrGr\hbar_{u}\hbar_{\theta }-\frac{1}{36}A_{3}A_{4}BrGrM^{2}\hbar_{u}\hbar_{\theta },$$

$$\begin{array}{l} D_{5}=+\frac{2}{3}A_{1}Br\hbar_{\theta }+\frac{1}{3}A_{1}A_{6}Br\hbar_{\theta }^{2}+\frac{1}{3Da}A_{1}^{2}Br\hbar_{1}\hbar_{2}-\frac{1}{3}A_{1}A_{2}BrF*\hbar_{u}\hbar_{\theta }+\frac{1}{6}A_{1}A_{4}BrGr\hbar_{u}\hbar_{\theta }-\\ \frac{1}{3}A_{3}BrM^{2}\hbar_{\theta }-\frac{1}{6}A_{3}A_{6}BrM^{2}\hbar_{\theta }^{2}-\frac{5}{6}A_{1}A_{3}BrM^{2}\hbar_{u}\hbar_{\theta }+A_{1}^{2}Br\hbar_{1}\hbar_{2}-\frac{11}{72Da}A_{1}A_{3}BrM^{2}\hbar_{u}\hbar_{\theta }+\\ \frac{13}{90}A_{2}A_{3}BrF*M^{2}\hbar_{u}\hbar_{\theta }-\frac{1}{12}A_{3}A_{4}BrGrM^{2}\hbar_{u}\hbar_{\theta }+\frac{1}{6}A_{3}^{2}BrM^{4}\hbar_{1}\hbar_{2}, \end{array}$$

$$D_{6}=-\frac{1}{20}A_{1}A_{4}BrGr\hbar_{u}\hbar_{\theta }+\frac{1}{60}A_{3}A_{4}BrGrM^{2}\hbar_{u}\hbar_{\theta },$$

$$\begin{array}{l} D_{7}=-\frac{2}{45}A_{1}^{2}Br\hbar_{1}\hbar_{2}+\frac{4}{45}A_{1}A_{2}BrF*\hbar_{u}\hbar_{\theta }+\frac{1}{30}A_{3}A_{6}BrM^{2}\hbar_{\theta }^{2}+\frac{7}{180Da}A_{1}A_{3}BrM^{2}\hbar_{u}\hbar_{\theta }+\\ \frac{1}{10}A_{1}A_{3}BrM^{2}\hbar_{u}\hbar_{\theta }-\frac{2}{45}A_{3}A_{2}BrF*M^{2}\hbar_{u}\hbar_{\theta }+\frac{1}{15}A_{3}BrM^{2}\hbar_{\theta }+\frac{1}{60}A_{3}A_{4}BrGrM^{2}\hbar_{u}\hbar_{\theta }-\\ \frac{1}{30}A_{3}^{2}BrM^{4}\hbar_{1}\hbar_{2}, \end{array}$$

$$\begin{array}{l} D_{8}=-\frac{1}{252}A_{3}A_{4}BrGrM^{2}\hbar_{u}\hbar_{\theta },\\ D_{9}=-\frac{1}{70}A_{1}A_{2}BrF*\hbar_{u}\hbar_{\theta }-\frac{1}{336}A_{1}A_{3}BrM^{2}\hbar_{u}\hbar_{\theta }+\frac{1}{140}A_{3}A_{2}BrF*M^{2}\hbar_{u}\hbar_{\theta },\\ D_{10}=-\frac{1}{1350}A_{3}A_{2}BrF*M^{2}\hbar_{u}\hbar_{\theta }. \end{array}$$
